# Adenovirus-mediated decorin expression induces cancer cell death through activation of p53 and mitochondrial apoptosis

**DOI:** 10.18632/oncotarget.20800

**Published:** 2017-09-08

**Authors:** A-Rum Yoon, JinWoo Hong, Chae-Ok Yun

**Affiliations:** ^1^ Department of Bioengineering, College of Engineering, Hanyang University, Seongdong-gu, Seoul 04763, Korea

**Keywords:** oncolytic adenovirus, decorin, p53, mitochondrial apoptosis

## Abstract

Decorin (DCN) is a small leucine-rich proteoglycan that plays an important role in the regulation of apoptosis, proliferation, intercellular contact, and cell migration. Here we have investigated the detailed mechanism of apoptotic cell death induced by DCN expression. A marked increase in cytotoxicity was observed for both DCN-expressing replication-incompetent (dE1/DCN) and -competent (dB/DCN) adenoviruses (Ads) compared to the corresponding control Ads. FACS and TUNEL assays revealed that the expression of DCN induced apoptotic cell death. Specifically, the expression and stability of p53 were increased by DCN. In addition, western blot data showed that DCN expression activated mitochondrial apoptosis by increasing the expression level of p53. Similarly, DCN-expressing oncolytic Ads induced a greater antitumor effect in a murine xenograft model compared with control Ads. Tissue staining and western blot data from *in vivo* experiments demonstrated significantly higher levels of apoptosis in tumor tissues from mice treated with DCN-expressing Ads compared to those treated with control Ads. Collectively, these data support that cell killing effect is enhanced with Ad-mediated DCN expression via the induction of p53-mediated mitochondrial apoptosis, which could be a valuable benefit for antitumor efficacy.

## INTRODUCTION

Oncolytic adenoviruses (Ads) have the ability to target, replicate in, and lyse cancer cells. However, due to the modest cytolytic activity of oncolytic Ads when used as an antitumor agent, the development of an improved therapy for cancer patients is necessary [1–3]. In order to achieve this goal, insertion of powerful therapeutic genes into oncolytic Ads has been explored [4]. Many oncolytic Ads armed with therapeutic genes have drastically attenuated oncogenic activities, such as proliferation [5, 6], angiogenesis [7–9], negative regulation of host immune system [10, 11], and remodeling of extracellular matrix (ECM) [12], making them highly efficacious in treating cancer. Moreover, the expression of these transgenes can be increased through selective replication of the oncolytic Ads in tumor cells, resulting in potent and targeted antitumor effect.

Decorin (DCN), a member of the small leucine-rich proteoglycan (SLRP) family [13, 14] is a regulator of the collagen fibrillogenesis component of ECM assembly [15]. DCN can bind with collagen and delay the lateral assembly of collagen fibrils, resulting in the reduced diameter of the fibrils [16]. DCN also affects the production of other ECM components by blocking the activity of transforming growth factor-β (TGF-β) [16]. In addition, DCN can inhibit the interactions between the ECM and cancer cells [17]. Furthermore, DCN affects ECM remodeling by induction of matrix metalloproteinase-1 [18]. Based on these critical biological roles of DCN on ECM assembly and rearrangement, we previously investigated the utility of DCN-expressing oncolytic Ad as an agent to enhance viral dispersion, and demonstrated that it enhanced the spread of replicating Ad *in vivo* [19]. Taken together, these reports highlight the potential of DCN to improve the efficacy of oncolytic Ad-mediated gene therapy

In addition to its prominent role as an ECM regulator, DCN also serves as a key signaling molecule that suppresses cancer cell growth, inhibits metastasis, and induces apoptosis in cancer cells. DCN expression is downregulated in hepatocellular, lung, and ovarian carcinomas [20–22]. Low expression level of DCN is associated with poor prognosis in invasive breast carcinoma, and with metastasis in a breast tumor model [23]. Furthermore, DCN tends to bind and downregulate several different receptor tyrosine kinases (RTKs), which are often overexpressed in cancer cells. One of the major roles of DCN is to signal through epidermal growth factor receptor (EGFR) and other members of the ErbB family of RTKs [16, 24], and through insulin-like growth factor receptor type I [25]. Furthermore, DCN has been reported to obstruct EGFR function and trigger apoptosis via caspase-3 activation, which leads to inhibition of *in vivo* cancer growth [27]. DCN has also been found to antagonize the MET proto-oncogene by increasing its intracellular degradation and by increasing cleavage of its extracellular domain [26].

Despite accumulating evidence which demonstrates the tumor suppressive effects of DCN on cancer cell growth and death, the detailed molecular mechanisms of DCN-mediated apoptosis remain poorly understood. In our current study, we hypothesized that the cancer inhibitory role of DCN is closely related to p53-mediated apoptosis. As such, we investigated the molecular mechanisms of DCN on the induction of p53-mediated apoptosis. We observed that DCN expression induced potent inhibition of cancer cell proliferation. Our findings also showed that DCN induced apoptosis, via a p53-dependent pathway, and accumulation of mitochondrial pro-apoptotic molecules in p53-positive cancer cells. Finally, DCN-expressing oncolytic Ad elicited potent therapeutic efficacy *in vivo*, which warrants its further testing in a clinical setting.

## RESULTS

### Enhanced cytopathic effect by DCN-expressing Ad

**Figure 1:**
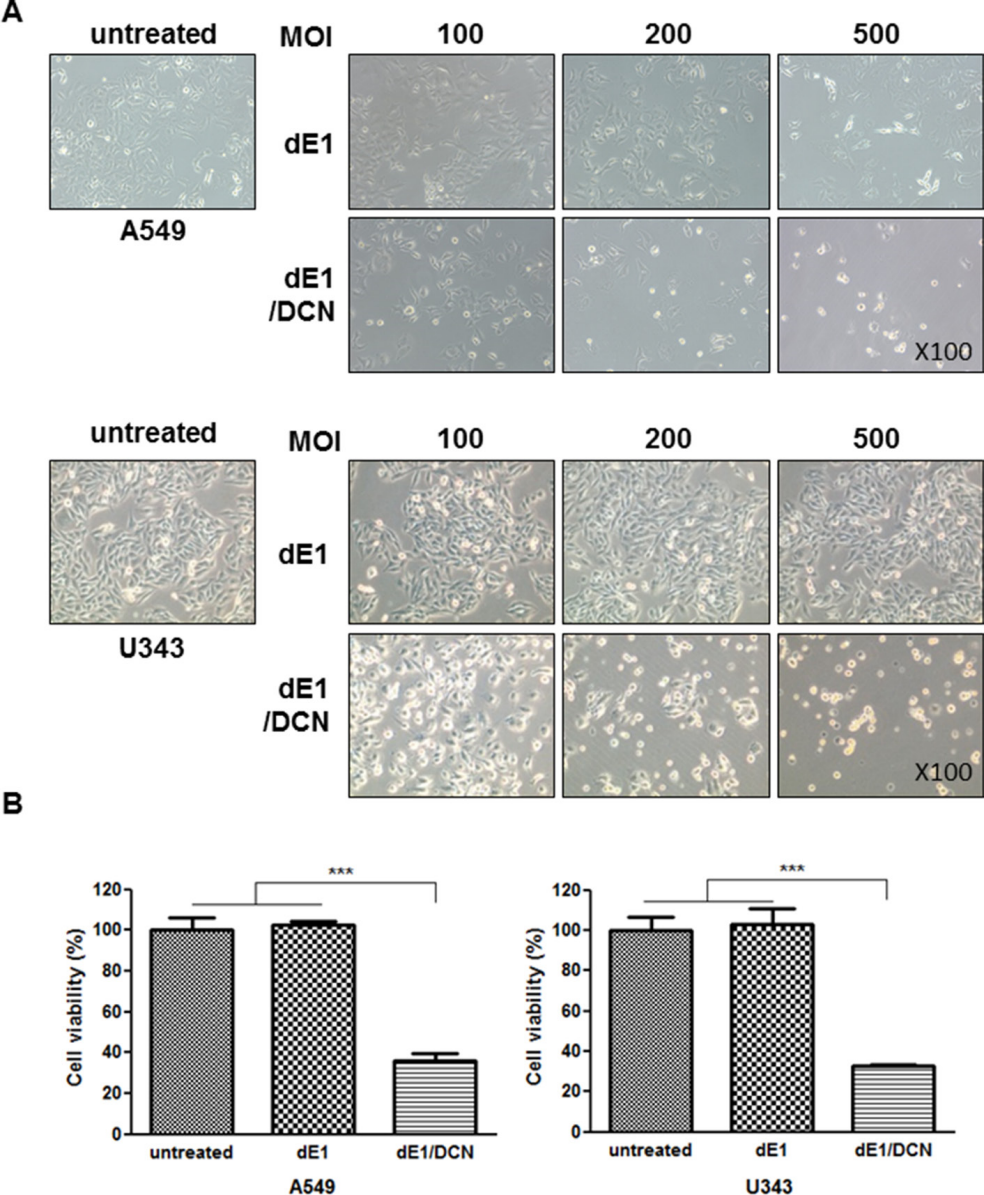
Enhanced cytopathic effect by DCN-expressing Ad (**A**) Morphological changes; A549 or U343 cells were transduced with dE1 or dE1/DCN at the indicated MOIs. At 48 hr post-infection, surviving cells were monitored and photos were taken using a microscope (×100). (**B**) MTT assay; cells were infected with dE1 or dE1/DCN. At 48 hr post transduction and, an MTT assay was performed to quantify the percentage of living cells. Replication-incompetent dE1 served as a negative control. Each cell line was tested at least three times and data shown are representative experiments performed in triplicate. Bars represent mean ± SD. ***P < 0.001.

We have previously reported that DCN-expressing Ads showed increased viral spreading and a greater cell-killing effect than control Ads lacking DCN [19]. Other studies have also suggested that DCN expression induced cancer cell killing and apoptosis [27, 28]. To confirm the cytopathic effect of DCN and its role in the induction of apoptosis, we visualized the morphology of U343 or A549 transfected with DCN-expressing Ad under a microscope and assessed their viability with an MTT assay. DCN-expressing replication-incompetent Ad (dE1/DCN) induced a potent cancer cell killing effect in a dose-dependent manner compared to the replication-incompetent control Ad (dE1) at all MOIs (Figure 1A). In agreement with the microscopic visualization, the MTT assay revealed that dE1/DCN significantly attenuated cell viability compared to dE1, which induced no cytopathic effect (Figure 1B; *P <* 0.001), indicating that expression of DCN induced cancer cell death. Furthermore, DCN-expressing oncolytic Ad (dB/DCN) elicited a greater cell-killing effect compared to the control oncolytic Ad (dB) (Supplementary Figure 1). Together, these results demonstrate that Ad-mediated DCN expression can induce cancer cell death and enhance the therapeutic efficacy of oncolytic Ads.

### Induction of apoptosis by DCN-expressing Ad

**Figure 2:**
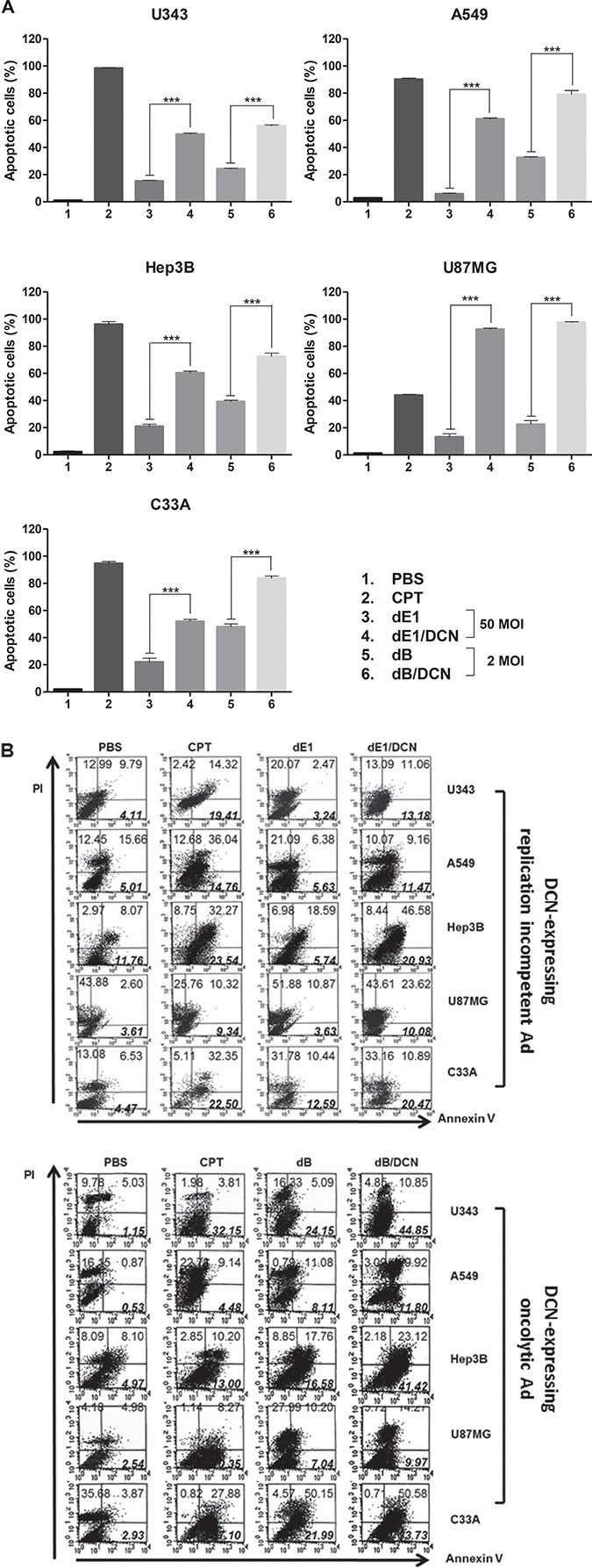
Induction of apoptosis by DCN-expressing Ad (**A**) *In vitro* TUNEL assay. The number of stained cells per 2,000 cells was counted. Each of the indicated values reflects the mean of three independent experiments, and is expressed as the percentage of apoptotic cells. The apoptotic cells were counted under 400× magnification in 10 selected fields. Each cell line was tested at least three times and data shown are representative experiments performed in triplicate. Bars represent mean ± SD. ***P < 0.001. (**B**) Analysis of Annexin-V/PI fluorescence; cells were infected with dE1, dE1/DCN, dB, or dB/DCN, or treated with CPT. At 2 days post-infection, cell were double-stained with Annexin V–fluorescein isothiocyanate (FITC)/propidium iodide (PI) and analyzed in a fluorescence-activated cell sorter (FACS). In each scatter plot, the percentage of early apoptotic cells (Annexin V–FITC (+)/PI (−), lower right quadrant) is bold and italicized.

Apoptosis, a programmed cell death, is one of the major cell death mechanisms [29]. Thus, we used a TUNEL assay to investigate whether DCN mediates cancer cell killing via induction of apoptosis. U343, A549, Hep3B, U87MG, or C33A cells were treated with either one type of replication-incompetent Ad (dE1 or dE1/DCN; MOI of 50) or one type of oncolytic Ad (dB or dB/DCN; MOI of 2) for 48 hr. Camptothecin (CPT), an apoptosis inducing agent, was used as a positive control. As shown in Figure 2A and Supplementary Figure 2, a greater percentage of cancer cells were undergoing apoptosis in all cells treated with DCN-expressing Ads compared to those treated with the respective control Ads. Transduction with dE1/DCN resulted in a higher apoptotic cell population compared to transduction with dE1 (U343 at 3-fold; A549 at 11-fold; Hep3B at 3-fold, U87MG at 7-fold; C33A at 2-fold; *P <* 0.001). Infection with dB/DCN also induced more apoptosis than dB in all cell lines (U343 at 2-fold; A549 at 2-fold; Hep3B at 2-fold, U87MG at 4-fold; C33A at 2-fold; *P <* 0.001). These results demonstrate that DCN expressed by Ads can induce apoptosis in several types of cancer cell lines.

Next, the extent of early apoptosis induced by DCN-expressing Ads was evaluated by Annexin V-PI double staining in U343, A549, Hep3B, U87MG, and C33A cells. CPT (1 μM) was used to induce apoptosis and to differentiate apoptotic cells from necrotic cells. Cells treated with dE1/DCN or dB/DCN showed a higher fraction of early apoptotic cells (Annexin V-positive/PI-negative) in all cell lines compared to controls (Figure 2B). Specifically, the early apoptotic fraction in dE1/DCN- or dB/DCN-treated U343 cells was 4.5- or 2.1-fold higher than in those cells treated with dE1 or dB, respectively. Similar patterns were observed in all other cancer cell lines tested. Cell cycle analysis also demonstrated that a higher population of cells were in the sub-G1 phase following treatment with DCN-expressing Ads in comparison to controls treated with non-DCN-expressing Ads (Supplementary Figure 3); this suggests that DCN also induces the later stage of apoptosis, which is associated with small and fragmented, genomic DNA. Taken together, these results indicate that DCN plays a role in the induction of apoptosis, leading to accelerated cancer cell death.

### Critical role of p53 in DCN-mediated induction of apoptosis

**Figure 3:**
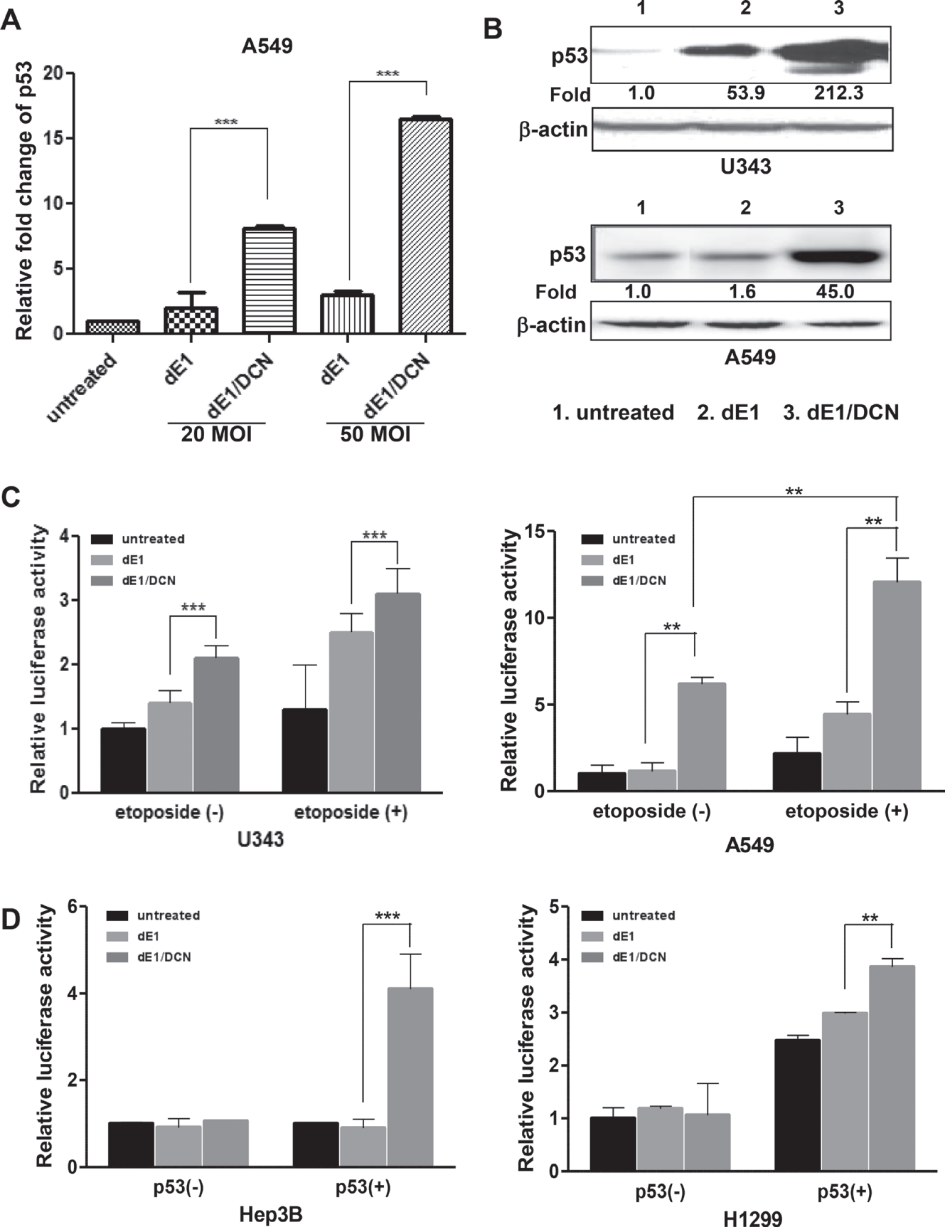
Augmented expression of p53 induced by DCN-expressing Ad (**A**) RT-qPCR analysis; total RNA was isolated from A549 cells infected dE1 or dE1/DCN, and RT-qPCR was performed using a p53 primer set. Each experiment was carried out at least three times and data shown are representative experiments performed in triplicate. Bars represent mean ± SD. ***P < 0.001. (**B**) Western blot; U343 and A549 cells were infected with dE1 or dE1/DCN at an MOI of 20. Cell lysates were probed with antibodies against p53. β-actin was used as an internal control to show equal protein loading. The expression levels of proteins were semi-quantitatively analyzed with the ImageJ software. (**C**) Luciferase assay; the DCN-mediated transcriptional activity of p53 was assessed in p53-positive U343 and A549 cells in the presence or absence of etoposide, a stimulator of apoptosis. Each experiment was carried out at least three times and data shown are representative experiments performed in triplicate. Bars represent mean ± SD. *P < 0.05, **P < 0.01, ***P < 0.001. (**D**) Luciferase assay; the DCN-mediated transcriptional activity of p53 was assessed in p53-null cancer cells (Hep3B and H1299) in the presence or absence of p53-expressing plasmid (pcDNA3.1-p53). Each experiment was carried out at least three times and data shown are representative experiments performed in triplicate. Bars represent mean ± SD. **P < 0.01.

Given that p53 has proven to be critical for the induction of apoptosis in tumor cells [30], we next investigated whether DCN-mediated induction of tumor cell apoptosis was regulated by the p53 signaling pathway. We first used qPCR analysis to examine whether DCN can enhance p53 mRNA expression in A549 cells. As shown in Figure 3A, p53 mRNA expression was significantly enhanced by dE1/DCN treatment in a dose-dependent manner (MOI of 20 and 50) compared to dE1 treatment (*P* < 0.001). Furthermore, dE1/DCN-treated cancer cells exhibited markedly higher levels of p53 protein expression than those treated with the control Ad dE1 (Figure 3B). These results suggest that DCN enhances p53 expression at both the transcriptional and translational levels.

Since an increase in p53-mediated transactivation of a pro-apoptotic gene has been reported to augment the induction of apoptosis [31], we next examined whether DCN-mediated induction of apoptosis was caused by its regulation of p53′s transactivation ability. Several cancer cell lines with different baseline expression levels of p53 were transfected with pG13-luc reporter plasmids harboring 13 putative p53 binding sites (5′-CCAGGCAAGTCCAGGCAGG-3′) in their proximal promoters, and then transduced with dE1/DCN for 48 hr. As shown in Figure 3C, dE1/DCN-treated cells in the absence of etoposide showed 1.6- and 5.3-fold higher luciferase activity in p53-positive U343 and A549 cells, respectively, compared to those treated with control Ad (*P <* 0.001 and *P* < 0.01), implying that DCN expression enhances the transactivation capacity of p53. Treatment with etoposide, which stimulates the apoptosis pathway through DNA damage-induced activation of p53, further increased the transactivation of p53 in A549 cells compared with those treated in the absence of etoposide (2.0-fold; *P <* 0.01), suggesting that DCN induces the transactivation activity of both preexisting endogenous p53 and new p53 generated by etoposide treatment. Furthermore, we also utilized a p53-deficient cancer cell lines (Hep3B and H1299) to isolate the effects of p53 expression on DCN-induced apoptosis. As shown in Figure 3D, luciferase activity, which measures the transactivation of target genes by p53 binding, was not altered by DCN expression in p53-null cells (Hep3B and H1299). Importantly, the transactivation capacity of p53 was restored when Ad-transduced p53-null cancer cells were transfected with p53-expressing plasmids Hep3B; *P* < 0.001, H1299; *P* < 0.01, highlighting the critical role of p53 expression in DCN-mediated apoptosis. Altogether, these findings demonstrate that DCN-mediated induction of apoptosis is p53-dependent, and it occurs through the transactivation of p53 as well as through an increase in the expression of p53 at both the transcriptional and translational level.

### Role of DCN in the post-translational modification of p53

**Figure 4:**
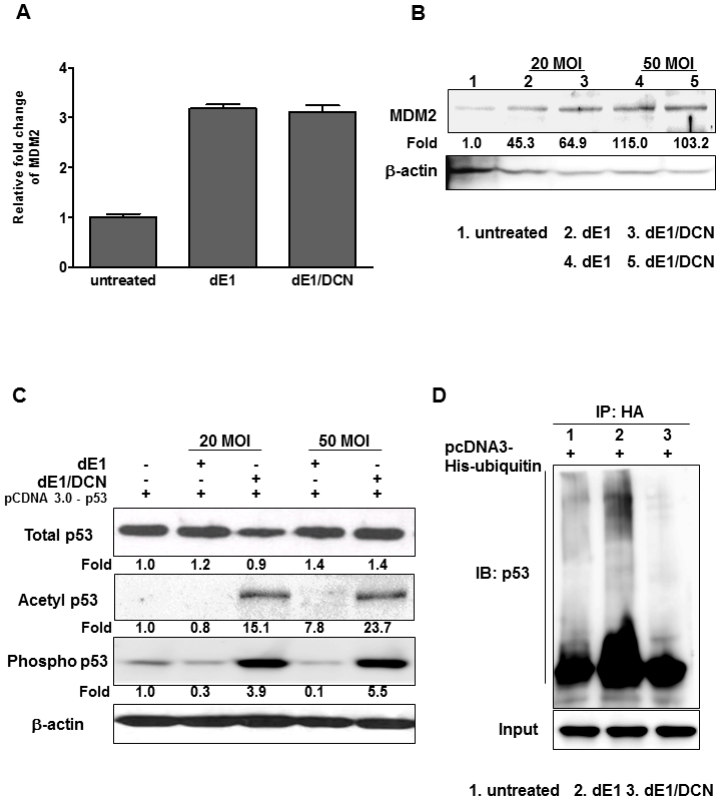
Changes in post-translational modification of p53 by DCN-expressing Ad (**A**) RT-qPCR analysis; total RNA was isolated from Hep3B cells infected with dE1 or dE1/DCN, and RT-qPCR was performed using an *Mdm2* primer set. Each experiment was carried out at least three times and data shown are representative experiments performed in triplicate. (**B**) Western blot; Hep3B cells were infected with dE1 or dE1/DCN at an MOI of 20 or 50. Cell lysates were probed with antibodies against MDM2. The expression levels of proteins were semi-quantitatively analyzed with the ImageJ software. (**C**) Western blot; Hep3B cells were infected with dE1 or dE1/DCN after transfection with the p53-expressing vector (pcDNA3.0). Cell lysates were probed with antibodies against p53, acetyl-p53 and phospho-p53. The expression levels of proteins were semi-quantitatively analyzed with the ImageJ software. (**D**) p53 ubiquitination assay. Cells were treated with mock, dE1, or dE1/DCN and His-tagged ubiquitin expression vectors in the presence of MG132. Cell lysates were co-immunoprecipitated with Ni-NTA beads and precipitates were analyzed by western blot using an anti-p53 antibody.

Among the post-translational modifications of p53, ubiquitination plays a key role in regulating both p53 stability and localization of p53 to the nucleus [32]. Mouse double minute 2 homolog (MDM2), a p53-specific E3 ubiquitin ligase, is the principal cellular antagonist of p53, and limits the p53 growth-suppressive function in unstressed cells [33]. As p53 regulates the cellular expression level of MDM2 and we wanted to assess whether DCN affect p53 function and stability through direct modulation of MDM2 expression in cancer cells, p53-null cell line Hep3B was used to compare the effect of dE1 and dE1/DCN on cellular MDM2 expression level without interference by endogenous p53. The results from qPCR and western blot analysis showed no significant changes in MDM2 expression after transduction with dE1 or dE1/DCN, suggesting that DCN-mediated p53-dependent apoptosis was not regulated by MDM2 (Figure 4A and 4B).

Certain post-translational modifications of p53, such as phosphorylation (on Ser15 residue) and acetylation (on Lys379 residue), improve p53 stability, accumulation, and activity, which influence the expression of p53 target genes [34]. To better elucidate the molecular mechanisms underlying DCN-mediated p53-dependent apoptosis, we investigated DCN's effect on p53 stabilization and activation by examining levels of acetyl- and phopho-p53 via western blot analysis. To differentiate any effects of endogenous p53 expression on post-translational modification of p53 from the DCN-mediated effects, a p53-null cancer cell line (Hep3B) was utilized. As seen in Figure 4C, transduction with dE1/DCN resulted in greater acetyl- and phospho-p53 expression than transduction with dE1 after an equal amount of an exogenous p53 expression vector (pCDNA-p53) was transfected into p53-null Hep3B cells. Furthermore, the expression of DCN decreased p53 ubiquitination compared with controls (untreated and dE1) (Figure 4D). Taken together, these results highlight that DCN induces the stabilization and activation of p53 in a manner that is independent of the MDM2-mediated ubiquitination of p53.

### Induction of mitochondrial control of apoptosis by DCN-expressing Ad

**Figure 5:**
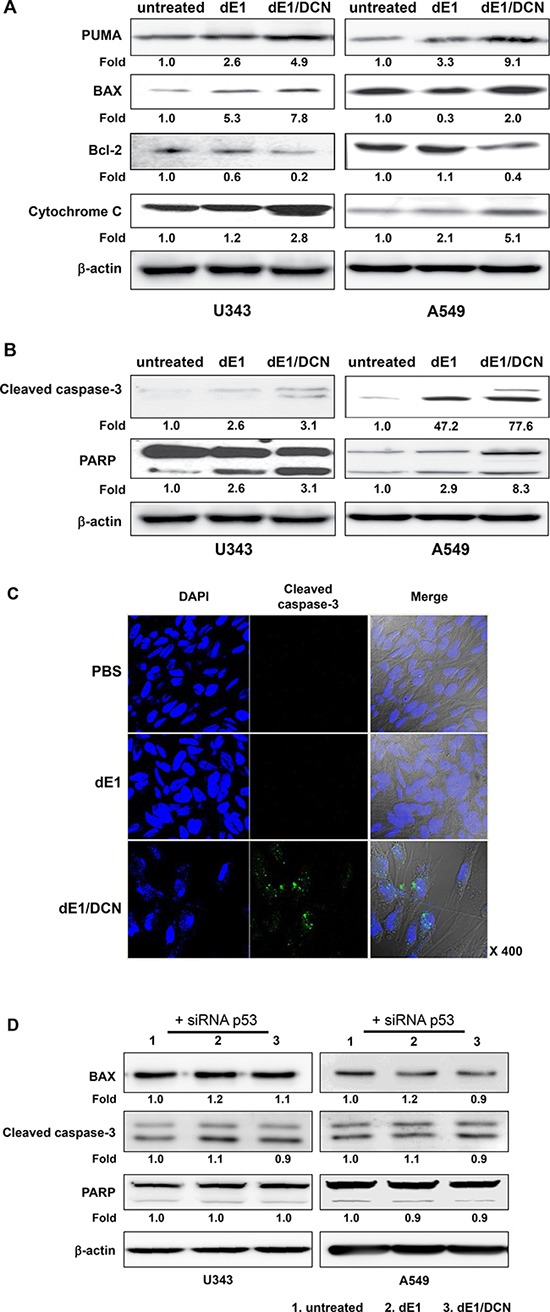
Activation of mitochondrial control of apoptosis by DCN-expressing Ad (**A**) Western blot; U343 and A549 cells were infected with dE1 or dE1/DCN at an MOI of 20. Cell lysates were probed with antibodies against PUMA, BAX, Bcl-2, and cytochrome C. β-actin was used as an internal control to show equal protein loading. The expression levels of proteins were semi-quantitatively analyzed with the ImageJ software. (**B**) Western blot; The U343 and A549 cells were infected with dE1, or dE1/DCN at an MOI of 20. Cell lysates were probed with antibodies against cleaved caspase and PARP. β-actin was used as an internal control to show equal protein loading. The expression levels of proteins were semi-quantitatively analyzed with the ImageJ software. (**C**) Immunocytochemistry; U343 cells were infected with dE1, or dE1/DCN at an MOI of 20. At 2 days post infection, fixed cells were probed with antibodies against cytochrome C using Alexa Fluor 488 anti-cleaved caspase-3 (green), and the nuclei were counterstained with DAPI (blue). (**D**) Western blot; U343 and A549 cells were infected with dE1 or dE1/DCN at an MOI of 20. Cell lysates were probed with antibodies against cleaved caspase and PARP. β-actin was used as an internal control to show equal protein loading. The expression levels of proteins were semi-quantitatively analyzed with the ImageJ software.

p53 induces apoptosis through regulation of Bcl-2-family-mediated mitochondrial permeability and stimulation of cytochrome C release [35]. In this study, we examined if DCN-mediated induction of apoptosis triggered mitochondrial control of apoptosis [36]. Several p53-mediated mitochondrial apoptosis proteins (PUMA, BAX, Bcl-2, and cytochrome C) were selected for western blot analysis [37]. As shown in Figure 5A, transduction with dE1/DCN induced greater expression of PUMA, BAX, and cytochrome C in both U343 and A549 cells when compared to transduction with the dE1 control Ad. Furthermore, the expression of Bcl-2, an anti-apoptotic protein, was markedly lower in the cells treated with dE1/DCN than in those treated with dE1. Together, these results demonstrate that DCN induces apoptosis by activating the p53-mediated mitochondrial apoptosis pathway.

We also confirmed that DCN-mediated induction of apoptosis occurred through the caspase-3 pathway, a critical promoter of apoptosis. As shown in Figure 5B, the expression level of both cleaved caspase-3 and PARP was higher in dE1/DCN-transduced cells compared to those transduced with dE1. In accordance with western blot analysis, immunocytochemical analysis revealed that dE1/DCN-treated cells exhibited increased expression of cleaved caspase-3 than those treated with dE1 (Figure 5C).

To assess whether DCN-mediated induction of mitochondrial apoptosis was dependent on p53, we examined the effect of transduction with DCN-expressing Ads on the expression levels of BAX, cleaved caspase-3, and PARP following transient silencing of p53 expression through pre-treatment with p53-targeted siRNA (Supplementary Figure 4). As shown in Figure 5D, after treatment with p53-specific siRNA, dE1/DCN-treated cells showed similar expression patterns for BAX, cleaved caspase-3, and PARP as dE1-treated negative control cells, in both the U343 and A549 cell line. These data imply that p53 expression is integral for DCN-mediated activation of both mitochondrial apoptosis and the caspase-3 signaling pathway that induces apoptotic cell death.

### Potent antitumor effect and survival benefit conferred by DCN-expressing oncolytic Ad

**Figure 6:**
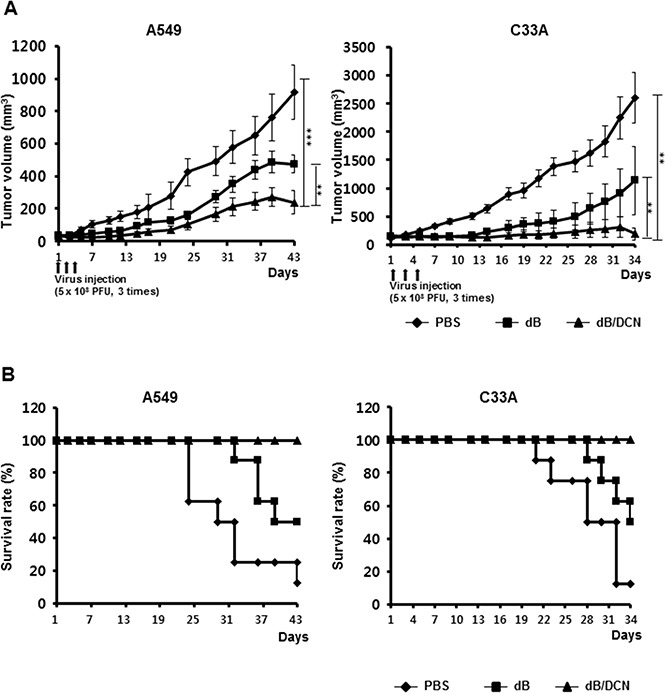
Suppression of the growth of established tumors and increased survival rate endowed by dB/DCN Tumors were grown subcutaneously in the abdomens of nude mice, and were subsequently injected directly with PBS, dB, or dB/DCN (**A**) Nude mice bearing tumors established by subcutaneous injection of C33A and A549 cells were treated three times with intratumoral injections of 5 × 10^8^ PFU/50 μl of Ad. Tumor growth was monitored at two or three day intervals until the end of the study. Values shown represent the mean ± SEM for eight animals per group. (**B**) Survival curve analysis. The percentage of surviving mice was determined by monitoring the death of mice over the given time periods.

We assessed the potential therapeutic efficacy of DCN-expressing oncolytic Ads (dB/DCN) in A549 and C33A human tumor xenografts. When the subcutaneously implanted tumors reached 6–6.5 mm in diameter, the mice were injected intratumorally every other day for a total of 3 times with dB or dB/DCN at a dose of 5 × 10^8^ PFU, or with PBS alone as a control. All xenograft tumors treated with dB/DCN exhibited significantly suppressed tumor growth when compared those treated with PBS or dB. As shown in Figure 6A, PBS-treated C33A tumors grew rapidly, and tumor size increased to an average size of 2603.96 ± 449.46 mm^3^ by day 34 following initial treatment. In marked contrast, dB- or dB/DCN-treated tumors reached an average size of 1135.24 ± 603.98 mm^3^ and 191.54 ± 104.61 mm^3^, showing a 56.4% and 92.6% growth inhibition, respectively, when compared with the PBS-treated tumors (*P <* 0.01 dB/DCN versus PBS or dB). Similar tumor growth inhibition was observed in A549 xenograft tumors treated with either dB or dB/DCN. In addition, survival rates were also significantly higher in mice treated with dB/DCN compared with dB-treated mice in all tested tumor xenograft models (C33A, A549: *P* < 0.01; Figure 6B). Throughout the course of the study, no systemic toxicity, such as diarrhea, weight loss, or cachexia, was observed. Taken together, these results suggest that DCN-expressing oncolytic Ad elicits potent inhibitory effects on tumor growth, resulting in prolonged survival.

### Enhanced apoptosis and viral replication induced by DCN-expressing oncolytic Ad

**Figure 7:**
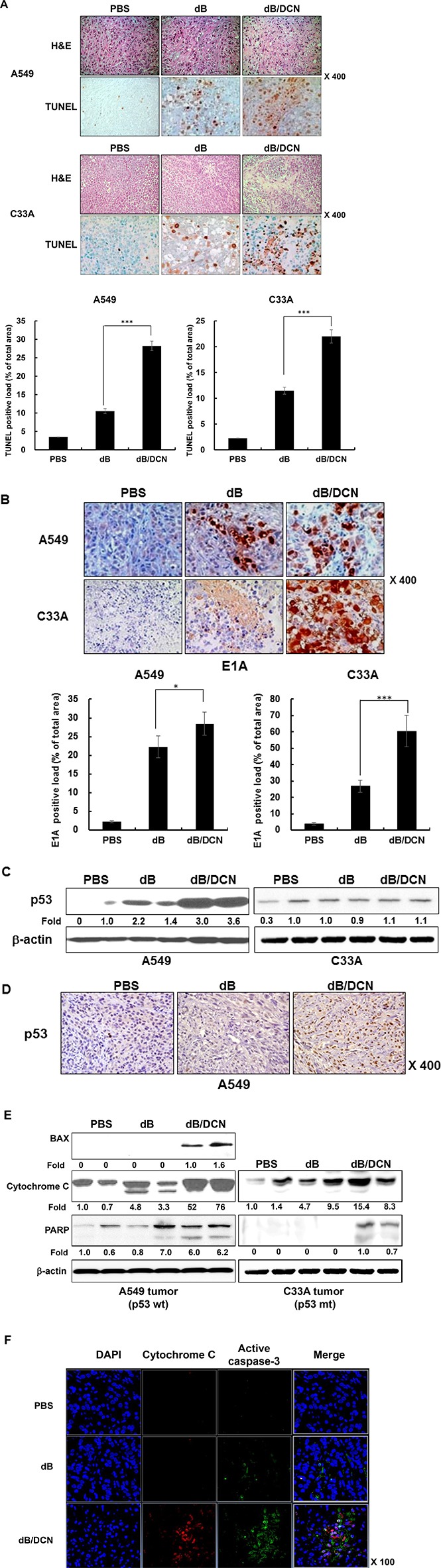
Histological analysis of tumor tissue injected with DCN-expressing Ad (**A**) PBS, dB, or dB/DCN was injected on day 0, 2, 4, into established C33A and A549 tumors in nude mice. Tumors were harvested on day 7 for histological analysis. H&E and TUNEL staining were performed in sections of PBS, dB, or dB/DCN-treated tumor tissue. The TUNEL positive spots were semi-quantitatively analyzed with the IHC Profiler Plugin in ImageJ software. ***P < 0.001 (**B**) PBS, dB, or dB/DCN was injected on day 0, 2, 4, into established C33A and A549 tumors in nude mice. Tumors were harvested on day 7 for histological analysis. Immunohistochemical staining of adenoviral E1A was performed in sections of PBS, dB, or dB/DCN-treated tumor tissue. The E1A positive spots were semi-quantitatively analyzed with the IHC Profiler Plugin in ImageJ software. *P < 0.05, ***P < 0.001 (**C**) Western blot; A549 and C33A tumors were treated with the oncolytic Ads (dB or dB/DCN). Tumor lysates were probed with antibodies against p53. β-actin was used as an internal control to show equal protein loading. The expression levels of proteins were semi-quantitatively analyzed with the ImageJ software. (**D**) Immunohistochemical staining of p53 was performed in sections of PBS, dB, or dB/DCN-treated tumor tissue. (**E**) Western blot; A549 and C33A tumor lysates treated with the oncolytic Ad dB or dB/DCN. Tumor lysates were probed with antibodies against BAX, cytochrome C, or PARP. The expression levels of proteins were semi-quantitatively analyzed with the ImageJ software. (**F**) Immunocytochemistry; immunocytochemical staining for cytochrome C and active capspase-3 was performed in sections of PBS, dB, or dB/DCN-treated tumor tissue.

To further characterize the antitumor effect and survival benefit bestowed by treatment with DCN-expressing oncolytic Ad, tumor tissues were examined by histological and immunohistochemical analysis. Tumors were harvested 3 days after the administration of the final dose of PBS, dB, or dB/DCN. Hematoxylin and eosin (H&E) staining revealed that the majority of remaining tumor masses treated with dB/DCN were necrotic, whereas necrotic lesions in dB-treated xenografts were only detectable in limited tumor regions (Figure 7A). The apoptotic tumor cell population was markedly higher in dB/DCN-treated tumor tissue when compared with tumor tissue treated with PBS or dB, demonstrating that the enhanced induction of apoptosis mediated by DCN contributed to the superior antitumor efficacy of dB/DCN. Furthermore, dB/DCN-treated tumors exhibited a markedly higher accumulation of Ad particles across a wider region of tumor than those treated with dB (Figure 7B), implying that DCN-expressing Ad replicates actively and spreads more efficiently within tumor tissue.

Furthermore, as shown in Figure 7C and 7D, there was a marked upregulation of p53 in both p53-positive A549 tumor lysate and tumor tissue from the mice treated with dB/DCN compared with mice treated with just dB. Enhanced expression of BAX, cytochrome C, and PARP was observed in dB/DCN-treated A549 tumor tissue compared to PBS- or dB-treated tumor tissue (Figure 7E). As shown in Figure 7F, treatment with dB/DCN also resulted in release of cytochrome C and activation of casepase-3 in A549 tumor tissue. Taken together, these results suggest that DCN can trigger apoptotic tumor cell death through activation of p53 and induction of mitochondrial apoptosis in a p53-positive tumor xenograft model. Of note, DCN treatment did not affect the expression level of p53 (Figure 7C) in C33A tumor lysates, yet elevated expression levels of cytochrome C, cleaved caspase-3, and PARP (Figure 7E and Supplementary Figure 5) were detected in these lysates. These results indicate that DCN can also induce apoptosis in p53-independent manner in p53-mutant cancer cells.

## DISCUSSION

Previous clinical trials have shown that the use of oncolytic Ads in treating cancer patients was not sufficiently efficacious as a therapy on its own, so more recent trials have focused on investigating the combination of oncolytic Ads with standard cancer therapy to ensure a sufficient therapeutic effect [38, 39]. In this study, we demonstrated that oncolytic Ads can elicit sufficient therapeutic efficacy on their own by expressing DCN, which induced both early and late stage apoptosis of tumor cells (Figures 2 and 6, Supplementary Figures 2 and 3). These results are in agreement with previous studies highlighting DCN's ability to induce apoptosis [19, 27, 40]. Although these studies have shown that DCN induces apoptosis via activation of caspase-3 [28], the upstream signaling of caspase-3 activation has not previously been examined. For the first time, we demonstrated that DCN induced cancer cell death through activation of p53-mediated mitochondrial apoptosis.

We observed that Ad-mediated expression of DCN in cancer cells elevated p53 expression at both the transcriptional and translational levels, which implied that DCN may induce apoptosis through the p53 signaling pathway (Figure 3A and 3B). Similar results were observed *in vivo*: p53 expression levels were significantly increased in tumor tissues treated with DCN-expressing oncolytic Ad in comparison to those treated with control Ad (Figure 7B and 7C). Further, DCN-mediated apoptosis was p53-dependent, as DCN-induced transactivation of p53 was critical for the induction of apoptotic cell death and no apoptosis was observed in the absence of p53 (Figure 3C and 3D). Importantly, DCN-mediated apoptosis exhibited similar characteristics to those shown by the well-known cytotoxic anticancer drug, cisplatin, which also induces strong apoptosis of tumor cells via transactivation of p53 [41]. Cisplatin-mediated transactivation of p53 can also increase transcription of proapoptotic genes, such as PUMA and caspase, whose expression are controlled by promoters with p53 binding sites [42]. In support of these findings, DCN expression increased the expression levels of p53-regulated proapoptotic proteins, such as PUMA, BAX, and cytochrome C in cancer cells both *in vitro* and *in vivo* (Figures 5A–5CA, 7E). Together, these findings suggest that controlled expression of DCN in tumor tissue can induce a potent apoptotic effect through a p53-dependent mechanism in a similar manner to cytotoxic chemotherapeutic.

The first evidence of link between DCN and p53 was reported using a DCN/p53 double knockout mice which demonstrated faster rate of tumor development than single knockout mice [43]. Yet, these preliminary findings failed to examine causal relationship between DCN and p53 in detail. Our current study shows that DCN increases p53 expression at both the transcriptional and translational levels (Figure 3A and 3B). These results in conjunction with reports by others, which demonstrated that p53 can transactivate DCN gene as DCN promoter contains p53-responsive element [44], suggests that DCN and p53 may synergistically enhance expression level of each other by forming a positive feedback loop. Although this pathway may support direct interaction between p53 and DCN, currently there are lack of concrete evidence as to how DCN directly regulates p53. Thus, future research should be carried out to examine DCN-mediated regulation of p53 in greater detail.

The robust DCN-mediated transactivation of p53 was caused by efficient phosphorylation and acetylation of p53, as DCN significantly elevated accumulation of post-translationally modified p53 in cancer cells (Figure 4C). These findings are in agreement with previous reports, which demonstrated the positive correlation between post-translational modification of p53, transcriptional activation of p53, and expression of apoptotic proteins such as PUMA, BAX, and cytochrome C [40, 45–47]. Indeed, transient silencing of p53 in cancer cells with p53-targeted siRNA nullified both DCN-mediated regulation of BAX and activation of the caspase-3 signaling pathway (Figure 5D). Together, these findings illustrate that DCN-mediated transactivation and post-translational modification of p53 can activate the mitochondria-dependent apoptosis pathway.

Another key component of p53-dependent apoptosis at the post-translational level is ubiquitination of p53. The post-translational interaction of p53 with MDM2 causes both ubiquitination and degradation of p53 [48]. In malignant tumors, MDM2 is often upregulated and overexpression of MDM2 confers resistance toward p53-mediated apoptosis of tumor cells [49]. Based on these reports, we hypothesized that DCN may co-modulate p53 and MDM2 expression level to induce apoptotic cancer cell death. Interestingly, our findings revealed that exogenous DCN did not significantly downregulate the MDM2 expression level in cancer cells (Figure 4B), yet enhanced activation and stabilization of p53 were observed in dE1/DCN-treated cells (Figure 4C and 4D). These findings illustrate that DCN-mediated stabilization of p53 and induction of apoptosis was resistant to negative regulation by MDM2, which is often overexpressed in cancer cells. Although MDM2 expression was not altered by DCN, DCN may downregulate other negative regulators of p53, such as β-catenin or c-JUN NH2-terminal kinase [48, 50, 51]. These candidates require further examination in future studies.

Primarily, p53 functions as nuclear protein. However, p53 can also function outside of the nucleus in a transcription-independent manner via protein–protein interactions [35]. Nuclear p53 can translocate to mitochondria and there interact with Bcl-2 family proteins, which leads to the inactivation of antiapoptotic proteins or activation of proapoptotic proteins [52]. In the current study we have found that Ad-mediated DCN expression regulated the expression level of Bcl-2 family proteins, such as PUMA, BAX, Bcl-2, suggesting that DCN may promote translocation of nuclear p53 to mitochondria where it can induce apoptotic cell death (Figure 5A).

The proapoptotic activity of the DCN-expressing oncolytic Ad, dB/DCN, was also evident *in vivo.* Tumors treated with dB/DCN showed extensive necrosis and a high level of apoptosis, resulting in potent tumor growth inhibition (Figures 6A, 6B and 7A). dB/DCN induced apoptosis by increasing p53 expression, caspase activation, and cytochrome C release in tumor tissue (Figure 7C to Figure 7E). These results are in good agreement with our *in vitro* results from Figure 4C and Figure 5A–5C, suggesting that oncolytic Ad-mediated expression of DCN can induce apoptosis of tumor cells *in vivo* through activation of the p53-dependent apoptotic pathway. Importantly, oncolytic Ad-mediated expression of DCN also enhanced dispersion of the Ad, and there was a positive correlation between the degree of apoptosis and viral spread in solid tumors (Figure 7B). These findings are in alignment with previous reports which demonstrated that the strong induction of apoptosis led to the enhanced dispersion of oncolytic Ad in tumor tissue via two mechanisms: (1) induction of apoptosis attenuated interstitial pressure and generated more free space within the tumor, which facilitated the diffusion and dispersion of the oncolytic Ad, and (2) apoptotic bodies can function as vesicles that help spread viral progenies to neighboring tumor cells [19, 53]. Alternatively, we have previously demonstrated that DCN expression degraded aberrant tumor ECM, which functions as a physical barrier to the dispersion and penetration of therapeutic Ads, by attenuating TGF-β expression levels [19]. Altogether, efficient viral distribution of DCN-expressing oncolytic Ads in tumor tissue, which greatly augments their antitumor efficacy, may occur through a bifunctional process involving the induction of apoptosis and ECM degradation.

In C33A (mutant type p53) tumor tissue, DCN increased caspase-3 activation (Supplementary Figure 5). This result suggests that DCN can also induce apoptosis in a p53-independent manner in p53-null or -mutant cancer cells. Consistent with our results, others have showed that DCN also can mediate induction of apoptosis through caspase-8 activation [27]. There are several hypotheses as to how DCN can induce p53-independent apoptosis in p53-deficient cell lines. One is that DCN can interacts with death receptors (DR), such as DR2 and DR3, to stimulate apoptotic signal transduction pathway [54, 55]. Another possibility is that DCN could induce p73, a pro-apoptotic protein that mimics many of the biological activities of p53 [56, 57], and promote apoptosis in p53-deficient cells. Lastly, several reports have suggested that exogenous DCN can increase p21 expression in a p53-independent manner to promote apoptosis of cancer cells [58]. Although these studies propose several probable mechanisms behind DCN-induced apoptosis in cancer cells, further studies are required to elucidate how p53-dependent and -independent apoptosis are intertwined and regulated by DCN in heterogenic tumor cell population.

In conclusion, oncolytic Ad-mediated expression of DCN is a promising strategy to induce potent apoptotic cell death of tumor cells through a p53-dependent pathway, by increasing the expression level and inducing stabilization of p53. Further, this robust induction of tumor cell apoptosis enables the oncolytic Ad to overcome the physical barriers of the tumor microenvironment, and ultimately enhances the dispersion of oncolytic Ads in solid tumors, resulting in potent tumor growth inhibition. Importantly, oncolytic Ad-mediated expression of DCN can emulate the strong apoptotic effect induced by conventional chemotherapeutics, making it a promising platform to supersede combination chemo/Ad therapy for the treatment of aggressive cancer.

## MATERIALS AND METHODS

### Cell lines and cell culture

All cell lines were cultured in Dulbecco's Modified Eagle's Medium (DMEM; Gibco BRL, Grand Island, NY, USA) supplemented with 10% fetal bovine serum (Gibco BRL), L-glutamine (2 mM), penicillin (100 IU/ml), and streptomycin (50 μg/ml). HEK293 (human embryonic kidney cell line expressing the Ad E1 region), the brain cancer cell line (U343; p53-positive), the non-small lung cancer cell lines (A549; p53-positive, H1299; p53-null), the cervical cancer cell line (C33A; p53-mutant), and the hepatocarcinoma cell line (Hep3B; p53-null) were purchased from the American Type Culture Collection (ATCC, Manassas, VA, USA).

### Adenoviral vectors

A DCN-expressing, E1-deleted replication-incompetent Ad vector (dE1/DCN) and a replication-competent oncolytic Ad vector expressing DCN (dB/DCN) were used in this study [19]. An E1-deleted replication-incompetent Ad vector (dE1) and a replication-competent oncolytic Ad vector (dB) were also prepared as controls. All viruses were propagated in HEK293 cells, and the purification, titration, and quality analysis of all Ads were performed as previously described [8], [59]. Viral particle (vp) numbers were calculated from measurements of optical density at 260 nm (OD_260_), where one absorbance unit is equivalent to 10^12^ vp per milliliter. Infectious titers (plaque forming unit (PFU) per milliliter) were determined using a limiting dilution assay in 293 cells. The vp-to-PFU ratio for dE1, dE1/DCN, dB, and dB/DCN were 57:1, 70:1, 22:1, and 43:1, respectively. After viral generation, PCR amplification and DNA sequencing were performed to verify viral structures.

### MTT assays

To evaluate the cytopathic effect of oncolytic Ads, 2–5 × 10^4^ cells were plated onto 24-well plates at about 70% confluence, and then treated with replication- incompetent Ad (dE1 or dE1/DCN) at a multiplicity of infection (MOI) of 50–200 or with replication-competent oncolytic Ad (dB or dB/DCN) at an MOI of 0.5–2. The cytotoxic effect of these vectors was monitored daily under a microscope. When cells exhibited signs of complete cell lysis, a 3-[4,5-dimethylthiazol-2-yl]-2,5-diphenyltetrazolium bromide (MTT) assay was carried out as previously described [9]. In brief, 200 μl of MTT (Sigma, St. Louis, MO, USA) in phosphate buffered saline (PBS; 2 mg/ml) was added to each well. After 4 hr of incubation at 37°C, the supernatant was discarded and the precipitate was dissolved in 1 ml of dimethylsulfoxide (DMSO). Plates were then read on a microplate reader at 540 nm.

### TUNEL assay

Apoptosis was analyzed using the terminal deoxynucleotidyl transferase-mediated deoxyuridine 5′-triphosphate-biotin nick end labeling (TUNEL) assay. U343 (5 × 10^4^), U87MG (5 × 10^4^), C33A (5 × 10^5^), Hep3B (4 × 10^5^), and A549 (5 × 10^4^) cells were plated onto a chamber slide one day prior to treatment, and then transduced with dE1 or dE1/DCN at an MOI of 50 or with dB or dB/DCN at an MOI of 2. Cells treated with camptothecin (CPT) at 1 μM were used as a positive control. At 48 hr after treatment with one of the Ads or CPT, cells were processed with an ApopTag kit (Oncor, Gaighersburg, MD, USA) according to the manufacturer's instructions for the detection of cleaved deoxyribonucleic acid *in situ*. The apoptotic cells were counted at 400 x magnification in 5 selected fields. More than 2,000 cells were counted in order to calculate the percentage of TUNEL-positive cells. For the detection of apoptosis *in vivo*, formalin-fixed tissue sections were deparaffinized in xylene, rehydrated in graded alcohol, and transferred to PBS. The slides were then treated with proteinase K (20 μg/ml) for 15 min, and endogenous peroxidase was blocked using 3% hydrogen peroxide in PBS for 10 min. The samples were washed three times in distilled water and incubated for 10 min at room temperature with TdT buffer. Excess TdT buffer was drained, and the samples were incubated for 1 hr at 37°C with terminal transferase and biotin-16-dUTP. The samples were then rinsed four times with TB buffer and incubated for 1 hr at 37°C with a 1:400 dilution of peroxidase-conjugated streptavidin. After incubation with streptavidin, the slides were rinsed with PBS and incubated for 5 min with 3, 3’-diaminobenzidine (DAB). The sections were then washed three times with PBS, counterstained with methyl green, and observed under a microscope.

### FACS analysis

Apoptosis and cell nuclear damage were determined by Annexin V- FITC and PI dual cell staining; stained cells were subsequently analyzed by flow cytometry [60]. Cells grown in 100 mm dishes were transduced with dE1 or dE1/DCN at an MOI of 20–50, or with dB or dB/DCN at an MOI of 0.5–5. As a positive control for the induction of apoptosis, cells were treated with CPT at 1 mM. After two days of treatment, cells were processed with the ApoAlert Annexin V-FITC apoptosis kit (Clontech, Palo Alto, CA, USA) according to the manufacturer's instructions. Apoptosis and nuclear cell damage were quantified on a fluorescence-activated cell sorter (FACS; Becton Dickinson, Sunnyvale, CA, USA) and data from 10,000 events were collected for further analysis.

### Quantitative real-time PCR of p53 mRNA expression

Total RNA was isolated from A549 cells using TRIzol reagent (Invitrogen, Carlsbad, CA, USA). cDNA was synthesized using 5 μg of total RNA, oligo dT (2.5 mM), and SuperScript^TM^ II Reverse Transcriptase (200 units) in 20 μl total volume using a reverse transcription kit (Invitrogen). Quantitative real-time PCR (qPCR) was performed using SYBR Green Master Mix (Applied Biosystems, Carlsbad, CA, USA). The following qPCR oligonucleotide primers sets were used: p53 forward (5′-CTGCTCAGATAGCGATGGTCTG-3′), p53 reverse, (5′-TTGTAGTGGATGGTGGTACAGTCA-3′), glyceraldehyde 3-phosphate dehydrogenase (GAPDH) forward (5′-CCCCTTCATTGACCTCAACTAC-3′), and GAPDH reverse (5′-TCTCGCTCCTGGAAGA TGG-3′).

### Western blot analysis

Cells were lysed in extraction buffer (10 mM Tris, pH 7.4; 100 mM NaCl; 1 mM EDTA; 1 mM EGTA; 1 mM NaF; 20 mM Na_4_P_2_O_7_; 2 mM Na_3_VO_4_; 1% Triton X-100; 10% glycerol; 0.1% SDS; and 0.5% deoxycholate) (Biosource, Carlsbad, CA, USA) in the presence of a protease inhibitor. Protein concentration was determined using the BCA Protein Assay (Bio-Rad, Hercules, CA, USA). Each sample (50–100 μg) was separated by sodium dodecyl sulfate polyacrylamide gel electrophoresis (SDS-PAGE). The gels were then electroblotted onto a polyvinylidene difluoride (PVDF) membrane. Membranes were blocked in PBS-T (137 mM NaCl, 2.7 mM KCl, 10 mM Na_2_HPO_4_, 2 mM KH_2_PO_4_, and 0.1% Triton X-100) containing 5% dry milk. After blocking, membranes were incubated with anti-decorin (AF143; R & D Systems, Minneapolis, MN, USA), anti-p53 (sc-53394, Santa Cruz Biotechnology, Santa Cruz, CA, USA), anti-phospho-p53 (Ser15-9286, Cell Signaling Technology, Beverly, MA, USA), anti-acety-p53 (Lys379-2570, Cell Signaling Technology), anti-MDM2 (sc-812, Santa Cruz Biotechnology), anti-PUMA (4976, Cell Signaling Technology), anti-BAX (556467, BD Pharmingen, TX), anti-Bcl-2 (2876, Cell Signaling Technology), anti-cytochrome C (sc-8385, Santa Cruz Biotechnology), anti-cleaved caspase-3 (9664, Cell Signaling Technology), anti-PARP (9542, Cell Signaling Technology), or anti-β-actin antibody (Sigma), and then incubated with either horse radish peroxidase (HRP)-conjugated anti-rabbit IgG (7074; Cell Signaling Technology) or HRP-conjugated anti-mouse IgG (7076; Cell Signaling Technology). The blots were developed with enhanced chemiluminescence (ECL; Pierce Biotechnology, Rockford, IL, USA). Finally, the expression levels of proteins were semi-quantitatively analyzed by ImageJ software (version 1.50b; U.S. National Institutes of Health, Bethesda, MD). To analyze ubiquitination of p53, Hep3B cells were treated with mock, dE1, or dE1/DCN and His-tagged ubiquitin expression vectors (Addgene, Cambridge, MA) in the presence of MG132 (Sigma). Cells were lysed, lysates were immunoprecipitated using Ni-NTA beads (Qiagen, Hilden, Germany), and then western blot analysis was performed.

### Transcriptional analysis of MDM2 or p53 gene expression

Luciferase activity assays were performed using pG13-luc or pGL2-HDM2 reporter plasmids in U343, A549, or H1299 cells infected with dE1 or dE1/DCN as well as pCDNA3-p53. Luciferase activity was measured as described in the manufacturer's manual (Luciferase assay kit; Promega, Madison, WI, USA) with a luminometer and normalized to the total protein concentration. Total protein concentration was measured with a BCA protein assay kit (Pierce Biotechnology, Rockford, IL, USA). Luciferase activity was expressed as total luciferase units per total protein contents. Experiments were carried out in duplicate and repeated at least three times.

### Antitumor effects of DCN-expressing oncolytic Ads *in vivo*

Tumors were implanted in the abdomens of 5 to 6 weeks-old male nude mice by subcutaneous injection of A549, C33A, or Hep3B cells (1 × 10^7^ in 100 μl of Hank's balanced salt solution (HBSS; Gibco BRL)). When tumors reached a size in the range of 70~100 mm^3^, animals were randomly assigned to one of three groups to receive PBS, dB, or dB/DCN (seven or eight mice per group). The first day of treatment was designated as day 0. Ads or PBS was administered intratumorally (5 × 10^8^ PFU in 50 μl of PBS) on days 1, 3, and 5. Tumor growth inhibition was assessed by taking measurements of tumor volume every 2 or 3 days. The volume of tumors was calculated from measurements of the major axis and minor axis using a caliper and the following formula: tumor volume = (minor axis in mm)^2^ × (major axis in mm) × 0.523. The percentage of surviving mice was determined by monitoring the tumor growth-related events (tumor size > 500 mm^3^ for A549, > 1500 mm^3^ for C33A) over a period of 43 days.

### Evaluation of tumor xenograft by histology and immunohistochemistry

For immunohistochemistry, slides with sections of mouse tumor tissue were deparaffinized in xylene and then hydrated through graded alcohols. Endogenous peroxidase in the sections was blocked with 3% hydrogen peroxide. The sections were incubated with CAS-BLOCK (Zymed, San Francisco, CA, USA) for 30 min at room temperature to block non-specific protein binding. Then the sections were incubated at 4°C overnight with the primary mouse anti-Ad E1A antibody, MAB8052 (Chemicon, Temecula, CA, USA), and then complexed with a biotinylated anti-goat secondary antibody (DAKO, Glostrup, Denmark). Mouse serum was added to the complexed antibodies to minimize potential interaction between any uncomplexed secondary antibody and endogenous mouse immunoglobulin in the tissue section. The antibody complex was then applied to the sections, which were incubated for 30 min, rinsed in buffer, and then incubated with streptavdin-peroxidase. We also used tumor tissue sections to observe the presence and location of the E1A antigen. The positive spots of TUNEL and E1A were semi-quantitatively assessed using the IHC Profiler Plugin for ImageJ software (U.S. National Institutes of Health).

### Statistical analysis

Data are expressed as the mean ± standard deviation (SD). Statistical significance was determined using a two-tailed Student's *t*-test or one-way ANOVA test (SPSS 13.0 software; SPSS, Chicago, IL, USA). *P*-values of less than 0.05 were considered to be statistically significant.

## SUPPLEMENTARY MATERIALS FIGURES AND TABLES



## References

[1] Liu TC, Galanis E, Kirn D (2007). Clinical trial results with oncolytic virotherapy: a century of promise, a decade of progress. Nat Clin Pract Oncol.

[2] Vile RG, Russell SJ, Lemoine NR (2000). Cancer gene therapy: hard lessons and new courses. Gene Ther.

[3] Kirn D, Martuza RL, Zwiebel J (2001). Replication-selective virotherapy for cancer: Biological principles, risk management and future directions. Nat Med.

[4] Yoon AR, Hong J, Kim SW, Yun CO (2016). Redirecting adenovirus tropism by genetic, chemical, and mechanical modification of the adenovirus surface for cancer gene therapy. Expert Opin Drug Deliv.

[5] Su C, Peng L, Sham J, Wang X, Zhang Q, Chua D, Liu C, Cui Z, Xue H, Wu H, Yang Q, Zhang B, Liu X (2006). Immune gene-viral therapy with triplex efficacy mediated by oncolytic adenovirus carrying an interferon-gamma gene yields efficient antitumor activity in immunodeficient and immunocompetent mice. Mol Ther.

[6] van Beusechem VW, van den Doel PB, Grill J, Pinedo HM, Gerritsen WR (2002). Conditionally replicative adenovirus expressing p53 exhibits enhanced oncolytic potency. Cancer Res.

[7] Kang YA, Shin HC, Yoo JY, Kim JH, Kim JS, Yun CO (2008). Novel cancer antiangiotherapy using the VEGF promoter-targeted artificial zinc-finger protein and oncolytic adenovirus. Mol Ther.

[8] Yoo JY, Kim JH, Kwon YG, Kim EC, Kim NK, Choi HJ, Yun CO (2007). VEGF-specific short hairpin RNA-expressing oncolytic adenovirus elicits potent inhibition of angiogenesis and tumor growth. Mol Ther.

[9] Yoo JY, Kim JH, Kim J, Huang JH, Zhang SN, Kang YA, Kim H, Yun CO (2008). Short hairpin RNA-expressing oncolytic adenovirus-mediated inhibition of IL-8: effects on antiangiogenesis and tumor growth inhibition. Gene Ther.

[10] Lee YS, Kim JH, Choi KJ, Choi IK, Kim H, Cho S, Cho BC, Yun CO (2006). Enhanced antitumor effect of oncolytic adenovirus expressing interleukin-12 and B7-1 in an immunocompetent murine model. Clin Cancer Res.

[11] He LF, Gu JF, Tang WH, Fan JK, Wei N, Zou WG, Zhang YH, Zhao LL, Liu XY (2008). Significant antitumor activity of oncolytic adenovirus expressing human interferon-beta for hepatocellular carcinoma. J Gene Med.

[12] Choi IK, Shin H, Oh E, Yoo JY, Hwang JK, Shin K, Yu DC, Yun CO (2015). Potent and long-term antiangiogenic efficacy mediated by FP3-expressing oncolytic adenovirus. International Journal of Cancer.

[13] Iozzo RV, Murdoch AD (1996). Proteoglycans of the extracellular environment: clues from the gene and protein side offer novel perspectives in molecular diversity and function. FASEB J.

[14] Iozzo RV (1999). The biology of the small leucine-rich proteoglycans. Functional network of interactive proteins. J Biol Chem.

[15] Reed CC, Iozzo RV (2002). The role of decorin in collagen fibrillogenesis and skin homeostasis. Glycoconj J.

[16] Goldoni S, Iozzo RV (2008). Tumor microenvironment: Modulation by decorin and related molecules harboring leucine-rich tandem motifs. Int J Cancer.

[17] Vogel KG, Paulsson M, Heinegard D (1984). Specific inhibition of type I and type II collagen fibrillogenesis by the small proteoglycan of tendon. Biochem J.

[18] Danielson KG, Baribault H, Holmes DF, Graham H, Kadler KE, Iozzo RV (1997). Targeted disruption of decorin leads to abnormal collagen fibril morphology and skin fragility. J Cell Biol.

[19] Choi IK, Lee YS, Yoo JY, Yoon AR, Kim H, Kim DS, Seidler DG, Kim JH, Yun CO (2010). Effect of decorin on overcoming the extracellular matrix barrier for oncolytic virotherapy. Gene Ther.

[20] Miyasaka Y, Enomoto N, Nagayama K, Izumi N, Marumo F, Watanabe M, Sato C (2001). Analysis of differentially expressed genes in human hepatocellular carcinoma using suppression subtractive hybridization. Br J Cancer.

[21] McDoniels-Silvers AL, Nimri CF, Stoner GD, Lubet RA, You M (2002). Differential gene expression in human lung adenocarcinomas and squamous cell carcinomas. Clin Cancer Res.

[22] Shridhar V, Lee J, Pandita A, Iturria S, Avula R, Staub J, Morrissey M, Calhoun E, Sen A, Kalli K, Keeney G, Roche P, Cliby W (2001). Genetic analysis of early- versus late-stage ovarian tumors. Cancer Res.

[23] Troup S, Njue C, Kliewer EV, Parisien M, Roskelley C, Chakravarti S, Roughley PJ, Murphy LC, Watson PH (2003). Reduced expression of the small leucine-rich proteoglycans, lumican, and decorin is associated with poor outcome in node-negative invasive breast cancer. Clin Cancer Res.

[24] Hu Y, Sun H, Owens RT, Wu J, Chen YQ, Berquin IM, Perry D, O'Flaherty JT, Edwards IJ (2009). Decorin suppresses prostate tumor growth through inhibition of epidermal growth factor and androgen receptor pathways. Neoplasia.

[25] Schonherr E, Sunderkotter C, Iozzo RV, Schaefer L (2005). Decorin, a novel player in the insulin-like growth factor system. J Biol Chem.

[26] Buraschi S, Pal N, Tyler-Rubinstein N, Owens RT, Neill T, Iozzo RV (2010). Decorin antagonizes Met receptor activity and down-regulates {beta}-catenin and Myc levels. J Biol Chem.

[27] Tralhao JG, Schaefer L, Micegova M, Evaristo C, Schonherr E, Kayal S, Veiga-Fernandes H, Danel C, Iozzo RV, Kresse H, Lemarchand P (2003). *In vivo* selective and distant killing of cancer cells using adenovirus-mediated decorin gene transfer. FASEB J.

[28] Seidler DG, Goldoni S, Agnew C, Cardi C, Thakur ML, Owens RT, McQuillan DJ, Iozzo RV (2006). Decorin protein core inhibits *in vivo* cancer growth and metabolism by hindering epidermal growth factor receptor function and triggering apoptosis via caspase-3 activation. J Biol Chem.

[29] Fesik SW (2005). Promoting apoptosis as a strategy for cancer drug discovery. Nat Rev Cancer.

[30] Bates S, Vousden KH (1999). Mechanisms of p53-mediated apoptosis. Cell Mol Life Sci.

[31] Momand J, Zambetti GP, Olson DC, George D, Levine AJ (1992). The mdm-2 oncogene product forms a complex with the p53 protein and inhibits p53-mediated transactivation. Cell.

[32] Dai C, Gu W (2010). p53 post-translational modification: deregulated in tumorigenesis. Trends Mol Med.

[33] Moll UM, Petrenko O (2003). The MDM2-p53 interaction. Mol Cancer Res.

[34] Sakaguchi K, Herrera JE, Saito S, Miki T, Bustin M, Vassilev A, Anderson CW, Appella E (1998). DNA damage activates p53 through a phosphorylation-acetylation cascade. Genes Dev.

[35] Mihara M, Erster S, Zaika A, Petrenko O, Chittenden T, Pancoska P, Moll UM (2003). p53 has a direct apoptogenic role at the mitochondria. Mol Cell.

[36] Reed JC (2000). Mechanisms of apoptosis. Am J Pathol.

[37] Vaseva AV, Moll UM (2009). The mitochondrial p53 pathway. Biochim Biophys Acta.

[38] Kirn D (2001). Clinical research results with dl1520 (Onyx-015), a replication-selective adenovirus for the treatment of cancer: what have we learned?. Gene Ther.

[39] Kirn D (2000). Replication-selective oncolytic adenoviruses: virotherapy aimed at genetic targets in cancer. Oncogene.

[40] Wu HJ, Liu Y, Xue AM, Wang SX, Chen Q, Guo MY, Zhang ZG (2008). [Pro-apoptotic effect of decorin on rat mesangial cells *in vitro*]. [Article in Chinese]. Zhonghua Bing Li Xue Za Zhi.

[41] Jiang M, Wei Q, Wang J, Du Q, Yu J, Zhang L, Dong Z (2006). Regulation of PUMA-alpha by p53 in cisplatin-induced renal cell apoptosis. Oncogene.

[42] Jiang M, Dong Z (2008). Regulation and pathological role of p53 in cisplatin nephrotoxicity. J Pharmacol Exp Ther.

[43] Iozzo RV, Chakrani F, Perrotti D, McQuillan DJ, Skorski T, Calabretta B, Eichstetter I (1999). Cooperative action of germ-line mutations in decorin and p53 accelerates lymphoma tumorigenesis. Proc Natl Acad Sci U S A.

[44] Katsuro Iwase TH (2015). Identification of the p53-Responsive Element in the Promoter Region of the Human Decorin Gene. Molecular Biology.

[45] D'Antoni ML, Torregiani C, Ferraro P, Michoud MC, Mazer B, Martin JG, Ludwig MS (2008). Effects of decorin and biglycan on human airway smooth muscle cell proliferation and apoptosis. Am J Physiol Lung Cell Mol Physiol.

[46] el-Deiry WS (1998). Regulation of p53 downstream genes. Semin Cancer Biol.

[47] Shangguan JY, Dou KF, Li X, Hu XJ, Zhang FQ, Yong ZS, Ti ZY (2009). [Effects and mechanism of decorin on the proliferation of HuH7 hepatoma carcinoma cells *in vitro*]. [Article in Chinese]. Xi Bao Yu Fen Zi Mian Yi Xue Za Zhi.

[48] Kubbutat MH, Vousden KH (1997). Proteolytic cleavage of human p53 by calpain: a potential regulator of protein stability. Mol Cell Biol.

[49] Koo T, Choi IK, Kim M, Lee JS, Oh E, Kim J, Yun CO (2012). Negative regulation-resistant p53 variant enhances oncolytic adenoviral gene therapy. Hum Gene Ther.

[50] Damalas A, Ben-Ze'ev A, Simcha I, Shtutman M, Leal JF, Zhurinsky J, Geiger B, Oren M (1999). Excess beta-catenin promotes accumulation of transcriptionally active p53. EMBO J.

[51] Fuchs SY, Adler V, Buschmann T, Yin Z, Wu X, Jones SN, Ronai Z (1998). JNK targets p53 ubiquitination and degradation in nonstressed cells. Genes Dev.

[52] Zhivotovsky B, Kroemer G (2004). Apoptosis and genomic instability. Nat Rev Mol Cell Biol.

[53] Kim J, Kim PH, Yoo JY, Yoon AR, Choi HJ, Seong J, Kim IW, Kim JH, Yun CO (2009). Double E1B 19 kDa- and E1B 55 kDa-deleted oncolytic adenovirus in combination with radiotherapy elicits an enhanced anti-tumor effect. Gene Ther.

[54] Kim K, Fisher MJ, Xu SQ, el-Deiry WS (2000). Molecular determinants of response to TRAIL in killing of normal and cancer cells. Clin Cancer Res.

[55] Evdokiou A, Bouralexis S, Atkins GJ, Chai F, Hay S, Clayer M, Findlay DM (2002). Chemotherapeutic agents sensitize osteogenic sarcoma cells, but not normal human bone cells, to Apo2L/TRAIL-induced apoptosis. Int J Cancer.

[56] Holcakova J, Ceskova P, Hrstka R, Muller P, Dubska L, Coates PJ, Palecek E, Vojtesek B (2008). The cell type-specific effect of TAp73 isoforms on the cell cycle and apoptosis. Cell Mol Biol Lett.

[57] Conforti F, Sayan AE, Sreekumar R, Sayan BS (2012). Regulation of p73 activity by post-translational modifications. Cell Death Dis.

[58] Zhang Y, Wang Y, Du Z, Wang Q, Wu M, Wang X, Wang L, Cao L, Hamid AS, Zhang G (2012). Recombinant human decorin suppresses liver HepG2 carcinoma cells by p21 upregulation. Onco Targets Ther.

[59] Kim J, Cho JY, Kim JH, Jung KC, Yun CO (2002). Evaluation of E1B gene-attenuated replicating adenoviruses for cancer gene therapy. Cancer Gene Ther.

[60] Yang WS, Park SO, Yoon AR, Yoo JY, Kim MK, Yun CO, Kim CW (2006). Suicide cancer gene therapy using pore-forming toxin, streptolysin O. Mol Cancer Ther.

