# Supplementary material for: Adenovirus-mediated decorin expression induces cancer cell death through activation of p53 and mitochondrial apoptosis
Source: Oncotarget. 2017 Sep 8;8(44):76666–85. doi: 10.18632/oncotarget.20800 (PMC5652734; doi:10.18632/oncotarget.20800)
Supplement: Supplementary file 1 [file oncotarget-08-76666-s001.pdf]

Adenovirus-mediated decorin expression induces cancer cell death through activation of p53 and mitochondrial apoptosis

SUPPLEMENTARY MATERIALS

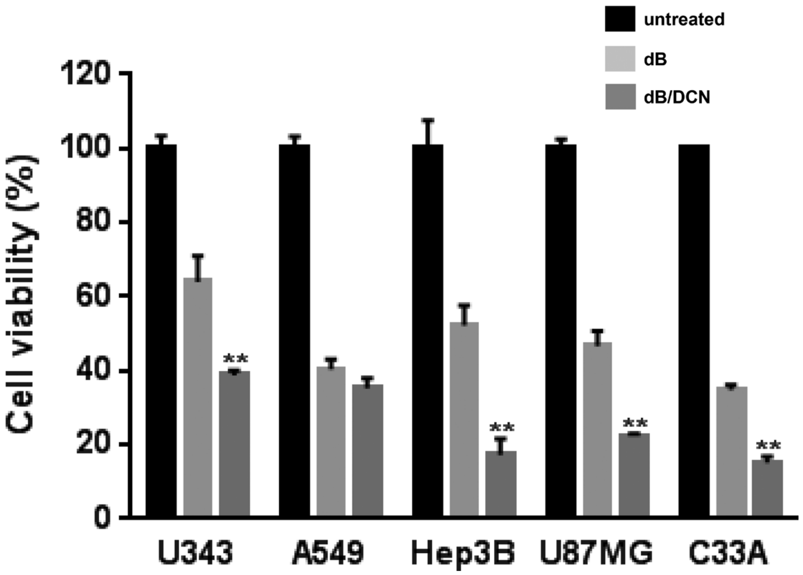

Supplementary Figure 1: Decorin-expressing Ads exhibit an enhanced cytopathic effect. MTT assay; cells were infected with dB or dB/DCN. At 2 days post-infection, an MTT assay was performed to quantify the percentage of living cells. Each cell line was tested at least three times and data shown are representative experiments performed in triplicate. Bars represent mean ± SD. $^{**}P < 0.01$.

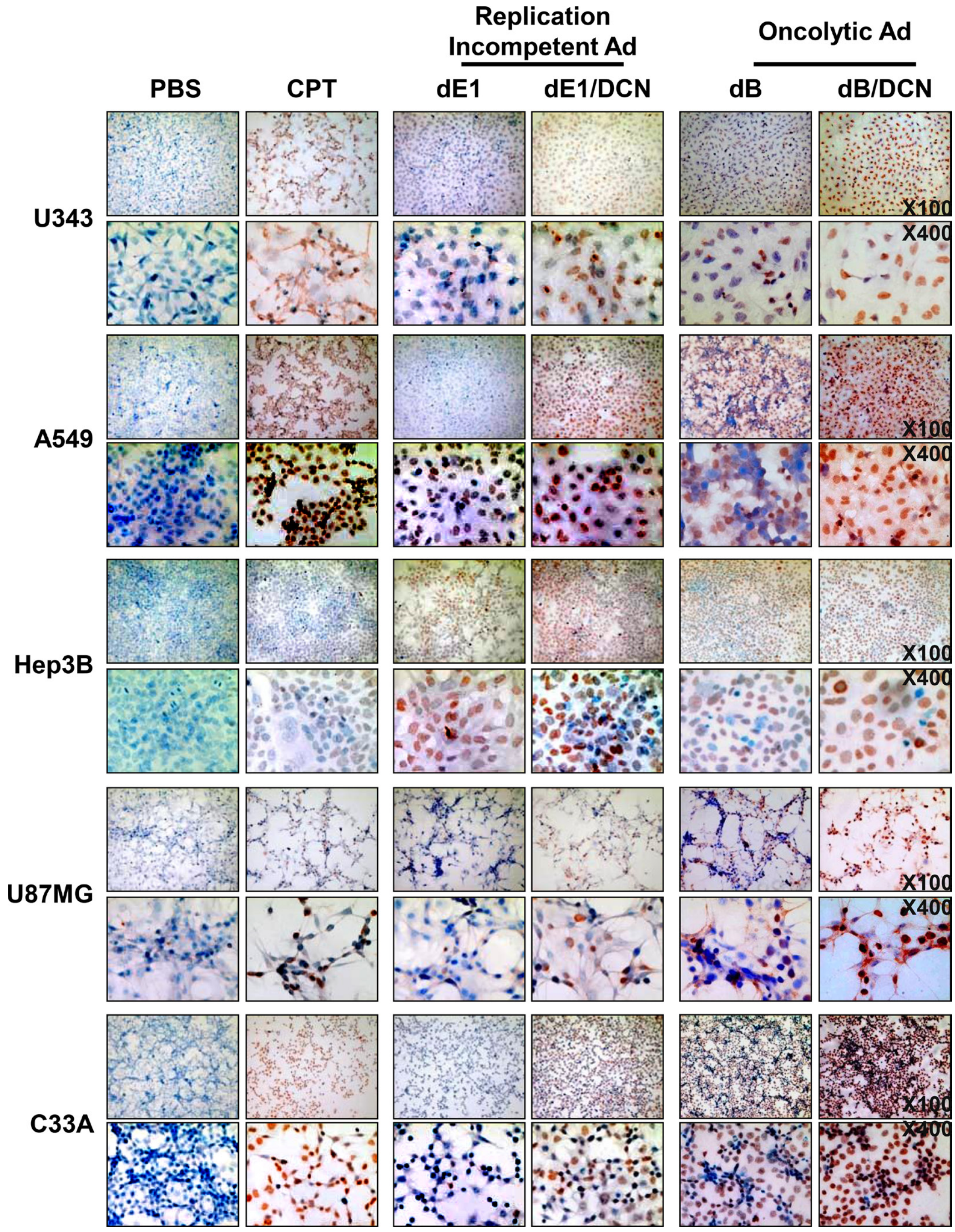

Supplementary Figure 2: Induction of apoptosis by decorin-expressing Ad. *In vitro* TUNEL assay; cells were infected with dE1, dE1/DCN, dB, or dB/DCN. At 48 hrs after infection with the Ad, cells were processed for the detection of cleaved deoxyribonucleic acid *in situ* using the terminal deoxynucleotidyl transferase-mediated deoxyuridine 5′-triphosphate-biotin nick end labeling (TUNEL) method. DNA strand breakage is indicated by the brown staining (counterstained with methyl green). Representative fields of three independent experiments are shown. Original magnification: 100× and 400×.

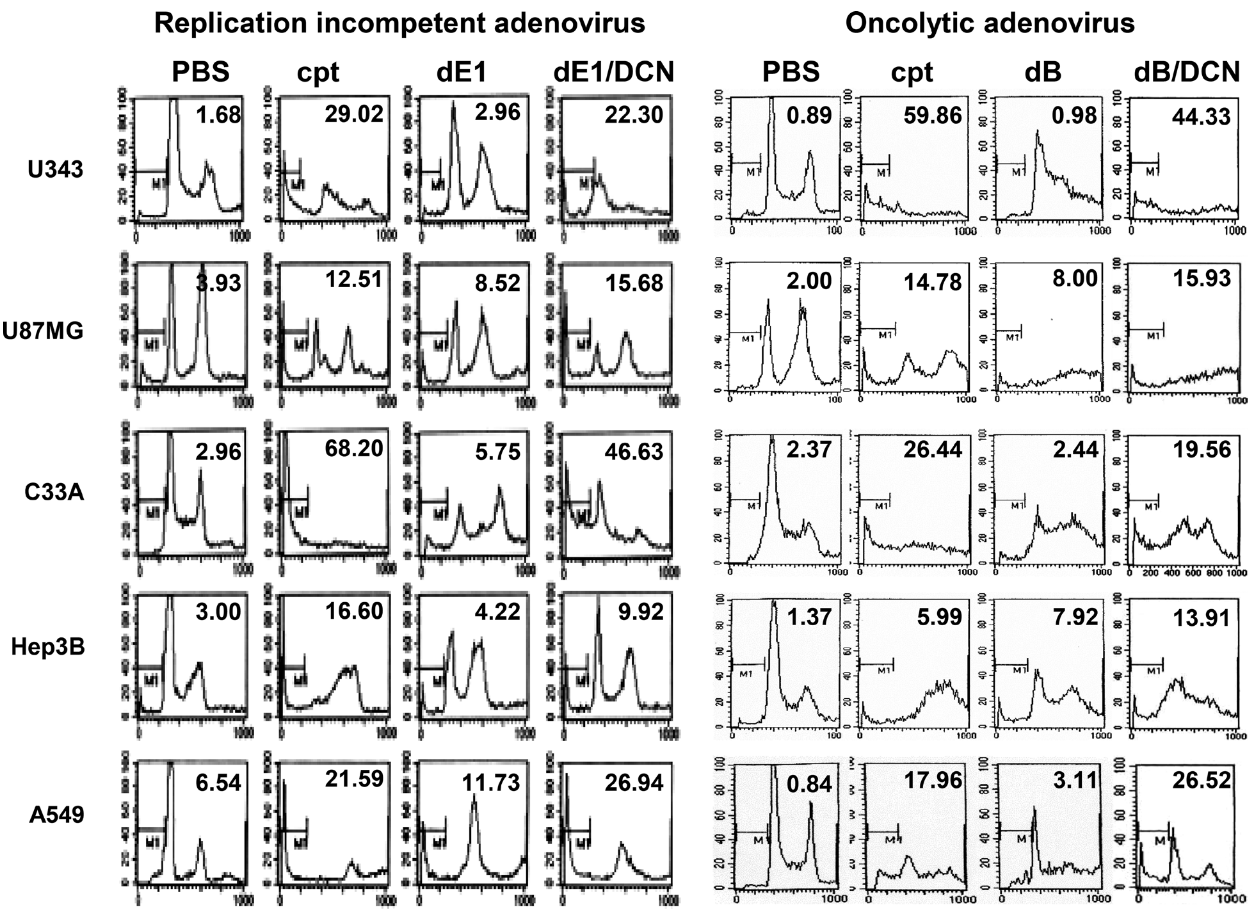

Supplementary Figure 3: Induction of apoptosis by decorin-expressing Ad. DNA content analysis; at 48 hr after treatment with 1 μM CPT, dE1, dE1/DCN, dB, or dB/DCN, cells were treated with RNase and stained with propidium iodide. The stained cells were then analyzed by flow cytometry.

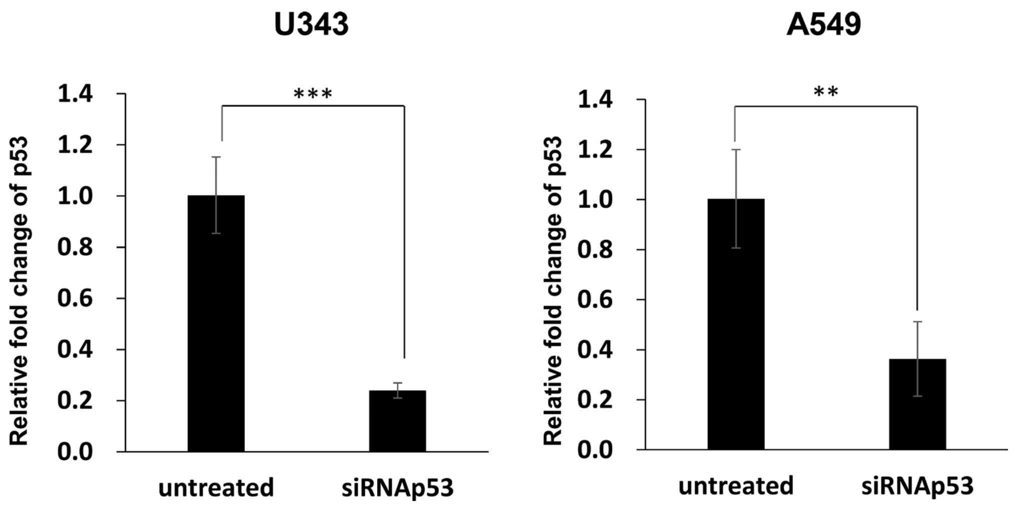

Supplementary Figure 4: Decreased p53 expression by p53-specific siRNA. Total RNA was isolated from U343 or A549 cells treated with siRNA against p53, and RT-qPCR was performed using a p53-specific primer set. Each experiment was carried out at least three times and data shown are representative experiments performed in triplicate. Bars represent mean ± SD. $^{**}P < 0.01$, $^{***}P < 0.001$.

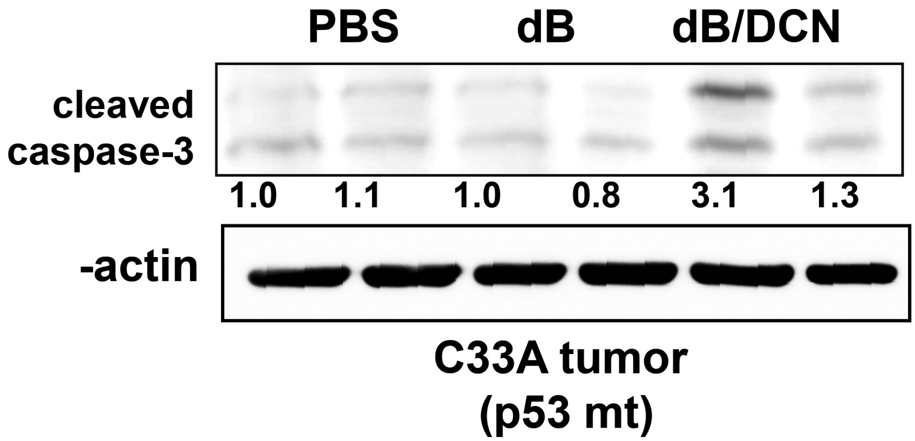

Supplementary Figure 5: Caspase cleavage in C33A tumor xenograft. Western blot analysis of C33A tumor lysates treated with the oncolytic Ads (dB or dB/DCN). Tumor lysates were probed with antibody against cleaved caspase-3. The expression levels of protein were semi-quantitatively analyzed with the ImageJ software.
